# The RISAP-study: a complex intervention in risk communication and shared decision-making in general practice

**DOI:** 10.1186/1471-2296-11-70

**Published:** 2010-09-22

**Authors:** Pia Kirkegaard, Adrian GK Edwards, Bo Hansen, Mette D Hansen, Morten SA Jensen, Torsten Lauritzen, Mette B Risoer, Janus L Thomsen

**Affiliations:** 1 School of Public Health, Dept. of General Practice, Aarhus University, Bartholins Alle 2, DK-8000 Aarhus C, Denmark; 2 Department of Primary Care and Public Health, Cardiff University, 2nd Floor Neuadd, Meirionnydd, Heath Park, Cardiff, CF14 4YS, UK; 3 Department of Community Medicine, Faculty of Health Sciences, University of Tromsø, Norway; 4 Institute of Public Health, Research Unit for General Practice, University of Southern Denmark, J.B. Winsløws Vej 9, DK-5000 Odense C, Denmark

## Abstract

**Background:**

General practitioners (GPs) and patients find it difficult to talk about risk of future disease, especially when patients have asymptomatic conditions, and treatment options are unlikely to cause immediate perceptible improvements in well-being. Further studies in risk communication training are needed. Aim:1) to systematically develop, describe and evaluate a complex intervention comprising a training programme for GPs in risk communication and shared decision-making, 2) to evaluate the effect of the training programme on real-life consultations between GPs and patients with high cholesterol levels, and 3) to evaluate patients' reactions during and after the consultations.

**Methods/Design:**

The effect of the complex intervention, based around a training programme, will be evaluated in a cluster-randomised controlled trial with an intervention group and an active control group with 40 GPs and 280 patients in each group.

The GPs will receive a questionnaire at baseline and after 6 months about attitudes towards risk communication and cholesterol-reducing medication. After each consultation with a participating high cholesterol-patient, the GPs will complete a questionnaire about decision satisfaction (Provider Decision Process Assessment Instrument). The patients will receive a questionnaire at baseline and after 3 and 6 months. It includes questions about adherence to chosen treatment (Morisky Compliance Scale), self-rated health (SF-12), enablement (Patient Enablement Instrument), and risk communication and decision-making effectiveness (COMRADE Scale). Prescriptions, contacts to the health services, and cholesterol level will be drawn from the registers.

In each group, 12 consultations will be observed and tape-recorded. The patients from these 24 consultations will be interviewed immediately after the consultation and re-interviewed after 6 months.

Eight purposefully selected GPs from the intervention group will be interviewed in a focus group 6 months after participation in the training programme.

The process and context of the RISAP-study will be investigated in detail using an action research approach, in order to analyse adaptation of the intervention model to the specific context.

**Discussion:**

This study aims at providing GPs and patients with a firm basis for active deliberation about preventive treatment options, with a view to optimising adherence to chosen treatment.

**Trial registration:**

ClinicalTrials.gov Protocol Registration System NCT01187056

## Background

Much attention has been given to how risk information should be communicated to patients with established conditions requiring treatment or specific surveillance strategies [1-3]. In prevention of disease, however, risk communication needs further elaboration; especially where preventive treatments are not likely to cause immediate perceptible improvements in well-being. This is a particular problem in the prevention of cardiovascular disease which is a major cause of morbidity, impaired quality of life and premature death worldwide. It has been argued that preventive treatment to lower the risk of cardiovascular disease is underused as a result of both general practitioners' low adherence to clinical guidelines and patients' low adherence to preventive treatment [4-6]. The issue therefore arises as to whether prevention of cardiovascular disease can be improved through enhanced communication.

High cholesterol is a well-defined risk factor for cardiovascular disease [7,8]. It is asymptomatic and usually detected by opportunistic screening in primary care. A change of life style is usually recommended to patients with high cholesterol. When this does not achieve sufficient reduction in the cholesterol level, cholesterol-reducing medication is usually suggested. Cholesterol-reducing medication has been shown to effectively reduce the risk of fatal heart disease [9]. The risk reduction depends on continuous (lifelong) medication. However, a review on prevention of cardiovascular disease has shown that 50% of all patients prescribed with cholesterol-reducing medication stop treatment within 6 months, with a further decline after one year. This decline is most prominent in patients with no symptoms of cardiovascular disease [10,11]. When patients do not comply with prescribed preventive medication for cardiovascular disease, the benefits will not be fully achieved [12,13].

Non-adherence to preventive medication may be due *inter alia *to inadequate knowledge about risk of disease, uncertainty about treatment benefits, and anxiety about side-effects [14]. In order for patient-centred care to be effective, it is important to support initiatives that ensure sufficient information about risks and benefits [15,16].

The choice to prioritise preventive treatment over watchful waiting should be taken collectively by the general practitioner (GP) and the individual patient [17]. Such co-operation is often complicated by contradictory perceptions of risk of disease and dangers associated with preventive treatment [18]. This is consistent with sociological and ethnographic studies showing that perceptions and deliberations of risk and danger are context-specific and embedded in knowledge traditions and complex socio-cultural relationships [19,20].

Shared decision-making (SDM) has been developed to enhance patient autonomy and engage patients in the process and responsibility of decision-making [21]. It includes a key component of risk communication [22]. SDM enhances patient involvement, though its benefits on patient-based outcomes, such as knowledge, anxiety, satisfaction and actual choice or adherence to chosen treatments (including choosing no treatment if relevant) are less clear [23,24]. SDM can be supported in different ways. Firstly, patient decision aids have been developed to facilitate patient-doctor communication about risk. Patient decision aids assist patients in making informed value-based choices by providing evidence-based information about a health condition, treatment options (including no-treatment), and potential benefits and harms, including risk numbers in different formats [25]. Secondly, training of healthcare professionals in shared decision-making, risk communication, and use of patient decision aids has been undertaken. Some studies have shown positive results in terms of patient satisfaction, patients' knowledge about risk, healthcare professional/patient-relationship, and patients' involvement in the medical decision [26,27] but this has been inconsistent in the literature to date [2].

There are a number of gaps in the evidence base. Training methods and training extent are often poorly described in published articles [28-30]. Few studies have determined effects of risk communication and/or decision aids on adherence to the chosen therapy [2]. Furthermore, only a few observational studies of doctor/patient risk communication have investigated real-life consultations [31-36]. Further studies in these areas of risk communication for disease prevention are needed as are studies about risk communication training for GPs and the use of decision aids. These studies need to develop and evaluate interventions that address a range of likely difficulties - in training and adoption of SDM and risk communication skills, use of decision aids with real patients, and follow-up of their effectiveness as patients try to adapt treatment plans to their everyday lives. By nature these are 'complex interventions' [37] and will benefit from multi-faceted evaluation [38].

## Objective

The objective of the RISAP study is 1) to systematically develop, describe and evaluate a complex intervention comprising a training programme for GPs in risk communication and shared decision-making, 2) to evaluate the effect of the training programme on real-life consultations between GPs and patients with high cholesterol, and 3) to evaluate the patients' reactions during and after the consultations and adherence to chosen treatment plans.

RISAP is an acronym for **Ris**k Communication in Gener**a**l **P**ractice.

The hypotheses are that the intervention group patients will have higher adherence to chosen treatment, have better self-rated health and fewer contacts with health services, have better satisfaction with treatment decision, and experience no increased anxiety, compared to the control group patients.

## Methods and design

### Participants

GPs in Region Central and Region North, Denmark, are invited to the study.

Patients are at least 18 years old, and have high cholesterol corresponding to a recommendation for cholesterol-reducing medication according to Danish clinical guideline for general practice [39]. The patients are recruited after their high cholesterol has been detected and when treatment options are to be discussed.

Patients with CVD or DM are excluded from the study, as are patients already receiving cholesterol-reducing medication and patients unable to speak and read Danish.

### Intervention

The intervention is a training programme for GPs in risk communication and shared decision-making. It consists of workshop sessions of 2×2 hours' duration and includes teacher-led discussions and GP role plays relating to risk communication, shared decision-making principles, and primary prevention of cardiovascular disease in general practice. In addition, the GPs will have training in the use of visual representations of risk and risk reduction, as well as a patient decision aid developed specifically for the training programme (please see "Development of the intervention" below).

The control group GPs will receive 2 hours of training in the primary care guideline for prevention of cardiovascular disease [39].

In both intervention group and control group, each workshop session will have 6-10 GPs led by two teachers from the teaching group (JT/BH/TL). The control group GPs will be invited to receive the full training programme after the conclusion of the study.

After participation in the workshop sessions, the GPs will return to their practices. When the GP identifies a patient with high cholesterol and is likely to suggest preventive pharmacological treatment, the GP will invite the patient to participate in the study. If the patient accepts participation, the GP will use the skills from the sessions (either intervention or control) during the consultation. Each GP will invite and recruit a minimum of 7 (consecutive) patients. Recruitment will be reimbursed with a fee of 300 DKK (35 £/40 €) per patient.

The study will be administered by three researchers and members of the RISAP project group. It will, however, also be administered by the participating GPs as they invite patients to the study.

### Development of the intervention

A preliminary draft for a training programme in risk communication and shared decision-making was developed by academic general practitioners and social scientists in the RISAP project group (PK, JT, MJ, AE, TL, BH, MR). The draft was based on literature review and experience from previous studies and entailed suggestions for improving ways to communicate about risk - including shared decision-making principles, visual representations of risk and risk reduction, decision support, and a patient decision aid.

61 GPs in Kolding, Region South, Denmark, were contacted by telephone by PK and BH, asking either the secretary or the GP permission to send a written invitation to a two-hour focus group interview. The invitations were sent directly to each of the GPs in the practices, with a note to return in an enclosed envelope to indicate willingness to participate (in Sept 2008). Participation was re-imbursed with a fee of 1400 DKK (165 £/188 €). 30 GPs returned the note, and 10 GPs (six male, four female) accepted participation on either of the two dates suggested in the written invitation, and two GPs (two female) offered participation on a later date. Average age was 48 years, ranging from 41 to 56 years.

The six GPs in the first focus group interview were first asked about their experiences with risk communication and decision-making with patients with high cholesterol. In the second half of the interview, the GPs were presented with the preliminary draft for the training programme. They were prompted to express their thoughts and ideas for further development from the preliminary draft. After the interview, these experiences, thoughts and ideas were categorised and interpreted, and members of the RISAP project group revised the preliminary draft accordingly. After the revision, the next two groups of GPs were interviewed, repeating the process of prompting and draft revision.

The GPs were asked to recruit at least one patient with high cholesterol deliberating cholesterol-reducing medication, for an individual interview with a researcher (PK). Four GPs recruited 12 patients, six male, six female. They were interviewed individually about their experiences with living with high cholesterol, treatment issues and with decision-making and risk communication with their GP. They were also presented with parts of the revised draft, i.e. the visual representations of risk and risk reduction, and the patient decision aid. They were prompted to express their thoughts and ideas for further development.

The data from the patient interviews were categorised and interpreted after the conclusion of the 12 interviews. The visual representations of risk and risk reduction and the draft decision aid were revised accordingly.

### Study Design and Randomisation

The effect of the complex intervention, based around a training programme for GPs, will be evaluated in a cluster-randomised controlled trial with an intervention group and an active control group [37,40,41].

Each participating practice will form a 'cluster'. Practices will be randomised to participate in either intervention or control group. The randomisation of the GPs is stratified according to single-handed practice or group practice, gender (male/female/both sexes) and age (average age of participating GPs if more than one GP from a group practice participate), and urban or rural practice. Randomisation will be undertaken by random number generation, and allocations by a program made by an independent statistician will be concealed from those implementing the intervention. Both GPs and patients will be informed that the study investigates cardiovascular risk management but the patients will be 'blinded' to which group they are in.

In each group, 12 consultations will be observed and tape-recorded by a researcher (PK). The consultations will be divided between 4 GPs, each with 3 patients. The GPs will be selected according to gender and age (1 male + 1 female GP ≥ 48 years, 1 male GP + 1 female GP < 48 years). The patients from these 2×12 consultations will be interviewed immediately after the consultation and re-interviewed after 6 months. The interview will be of approx. 45 minutes duration. The tape-recorded consultation will be played back during the interview with the patient, using the Think Aloud-method [42,43]. The observations during the consultation will be written down as ethnographic field notes [44]. The aim is to make a rich and detailed 'thick' description [45] of the consultation. The patient interviews after the consultations will focus on the patients' immediate reactions to risk communication and decision-making in the consultation, using a semi-structured interview guide with open questions and play back of the tape-recorded consultation. The patients will be re-interviewed after 6 months.

Eight GPs from the intervention group will be interviewed in a focus group 6 months after participation in the workshop sessions. They will be selected according to gender and age (2 male + 2 female GP ≥ 48 years, 2 male GP + 2 female GP < 48 years). The purpose of the focus group interview is to get feedback on the intervention process and the training programme, with a particular view to examining their assessment of the risk communication tools developed for the intervention, to inform future implementation strategies.

The process and context of the RISAP-study will be investigated in detail using an action research approach [46-48]. It includes 'thick' ethnographic descriptions of the research process in order to describe and analyse the continuous critical reflection in the project group on research choices, adaptation of intervention model to the specific context, and conscious and pragmatic performance strategies in scientific and political communities and networks [49-51].

### Outcomes

The primary patient outcome is adherence to treatment choice. Secondary patient outcomes are self-rated health, anxiety, satisfaction with decision, enablement, satisfaction with communication, satisfaction and confidence in decision, and number of contacts to health services.

The primary GP outcome is satisfaction with decision.

Secondary GP outcomes are GP attitudes towards risk communication and towards preventive treatment of cardiovascular disease, patient involvement in decision-making, and use of risk communication tools.

### Outcome measurement

The patients will receive a questionnaire at baseline and after 3 and 6 months. It includes questions from validated scales: the Morisky Compliance Scale is used to assess adherence to chosen treatment, defined as 80% of the scheduled treatment taken as prescribed [52-54]. Self-rated health will be measured with questions from SF-12 [55-64], and patient enablement will be measured with the Patient Enablement Instrument [65]. Risk communication and decision-making effectiveness will be measured using the COMRADE scale [66]. In addition, the questionnaire will include questions on the patients' socio-demographic circumstances.

Prescriptions, contacts to the health services, and cholesterol level will be drawn from the register of the National Health Service of Denmark at baseline and at 6 months. The cholesterol level will be measured only on the GP's own initiative.

The GPs will receive a questionnaire at baseline and after 6 months about attitudes towards risk communication and cholesterol-reducing medication. After each consultation with a participating high cholesterol-patient, the GPs will complete a questionnaire about decision satisfaction with questions from the Provider Decision Process Assessment Instrument [67]. In addition, the GP will be asked to identify which, if any, risk communication tools were used during the consultation.

### Sample size

A simulated power calculation shows that a trial with 40 practices in each group (at least 1 GP per practice and at least 7 patients with high cholesterol per GP) ensures a statistical power of 90% for detecting a 10% difference in lack of adherence (from 20% to 10%) at a significance level of 5%. The patients' adherence rates are assumed to vary from GP to GP according to a normal distribution on a logarithmic scale. The variance of the latent normal distribution is determined by assuming that 95% of the patients adherence levels within each GP vary within the intervals 0.08 to 0.50 (active control group) and 0.04 to 0.25 (experimental group).

### Data analysis

Descriptive statistics will be generated for all variables. All analyses will be intention to treat. Data concerning adherence will be analysed by multivariate binomial regression with a latent variable with a level for each practice. Multivariate logistic regressions models will be used to determine variables independently associated with the outcome variable for questionnaire data. Missing responses will be excluded from the analysis. A probability level of p < 0.05 based on two-sided tests is considered statistically significant.

The qualitative research process will be iterative, alternating between data production, analysis, and theory. Informal analysis has been conducted in the developmental stage of the study and continues in a kind of funnel structure of "progressive focusing" [51]. The data from the interviews will be transcribed verbatim, and these transcriptions and written field notes will undergo a thematic analysis in which key patterns will be identified and grouped into themes [68]. The core themes and their meanings and interrelations will be explored and interpreted, using appropriate formal theories [69].

## Discussion

Preventive treatment is fraught with uncertainty about the "true state" of health of asymptomatic patients at risk of becoming seriously ill. Both GPs and patients find it complicated to talk about the risk numbers and risk formats that may be a substantial part of the deliberation. This study aims at providing GPs and patients with a firm basis for active deliberation about preventive treatment options, with a view to optimising adherence to chosen treatment. The study results will provide a basis for recommendations about ways to communicate risk and facilitate shared decision-making during general practice consultations. The interviews and observations will allow the researchers in the project group to describe and support the learning processes during the training sessions in the complex intervention. This gives the opportunity to make the content, processes, methods and challenges as transparent as possible in the reporting of the results, adding much needed experience gathering about the possible 'active ingredients' of complex interventions in risk communication and shared decision-making. This will be interpreted to inform future implementation strategies for similar interventions in primary care.

## Ethical aspects

The study will be conducted according to the Helsinki Declaration. It has been notified to the Danish Data Protection Agency and collection of data will be handled according to their guidelines. The study is exempted from obligation of notification for the Scientific-ethical committee (pursuant to § 8 [3] of the rules of the committee) but follows the ethical code of American Anthropological Association [70]. It has been registered at ClinicalTrials.gov: NCT01187056.

## Competing interests

The authors declare that they have no competing interests.

## Authors' contributions

PK contributed to study design and development of intervention. She interviewed and analysed the results from the focus groups, and drafted the study protocol. AE contributed to study design, development of intervention, and interpretation of findings from focus groups. He also contributed to drafting of the study protocol. BH developed questionnaires and contributed to study design.

MDH completed the questionnaires, contributed to study design and commented on the study protocol. MJ contributed to study design and questionnaires. TL contributed to study design and development of intervention, and commented on the study protocol. MR supervised the qualitative part and commented on the study protocol. JT initiated, designed, and leads the study, developed the intervention, and contributed to the drafting of study protocol.

All authors read and approved the final manuscript.

## Authors' information

Pia Kirkegaard (PK):

PhD-student, Anthropologist

School of Public Health, Dept. of General Practice

Aarhus University

Bartholins Alle 2, 8000 Aarhus C

Denmark

E-mail: pia.kirkegaard@alm.au.dk

Adrian GK Edwards (AE):

B.Med.Sci MB BS MRCP MRCGP PhD, Research Professor in General Practice

Department of Primary Care and Public Health

Clinical Epidemiology Inter-disciplinary Research Group

Cardiff University

2nd Floor Neuadd Meirionnydd, Heath Park, Cardiff, CF14 4YS UK.

E-mail: edwardsag@cf.ac.uk

Bo Hansen (BH):

Clinical assistant, MD

School of Public Health, Dept. of General Practice

Aarhus University

Bartholins Alle 2, 8000 Aarhus C

Denmark

E-mail: bo.hansen@alm.au.dk

Mette D Hansen (MH):

Research assistant, Master of Health Science

School of Public Health, Dept. of General Practice

Aarhus University

Bartholins Alle 2, 8000 Aarhus C

Denmark

E-mail: mdh@alm.au.dk

Morten SA Jensen (MJ)

Clinical Assistant, PhD, MD

School of Public Health, Dept. of General Practice

Aarhus University

Bartholins Alle 2, 8000 Aarhus C

Denmark

E-mail: msa.jensen@gmail.com

Torsten Lauritzen (TL)

Professor, DMsc

School of Public Health, Dept. of General Practice

Aarhus University

Bartholins Alle 2, 8000 Aarhus C

Denmark

E-mail: tl@alm.au.dk

Mette B Risoer (MR):

Senior researcher, PhD, Anthropologist

Department of Community Medicine

Faculty of Health Sciences

University of Tromsø

9037 Tromsø

Norway

E-mail: mette.bech@uit.no

Janus L Thomsen (JT)

Associate Professor, MD, PhD, project leader

School of Public Health, Dept. of General Practice

Aarhus University

Bartholins Alle 2, 8000 Aarhus C

Denmark

&

Institute of Public Health, Research Unit for General Practice

University of Southern Denmark

J.B. Winsløws Vej 9

5000 Odense C

Denmark

E-mail: jlt@dadlnet.dk

## Pre-publication history

The pre-publication history for this paper can be accessed here:

http://www.biomedcentral.com/1471-2296/11/70/prepub
